# Long-term Outcome of Total Femur Replacement

**DOI:** 10.5704/MOJ.2307.004

**Published:** 2023-07

**Authors:** AL Adzhar, WI Faisham, W Zulmi, WS Azman, Y Sahran, AH Syurahbil, MZ Nor-Azman

**Affiliations:** 1 Department of Orthopaedics, Universiti Sains Malaysia, Kubang Kerian, Malaysia; 2 Department of Orthopaedics, Prince Court Medical Centre, Kuala Lumpur, Malaysia; 3 Department of Plastic Surgery, Universiti Sains Malaysia, Kubang Kerian, Malaysia

**Keywords:** total femur replacement, long-term outcome, limb salvage surgery

## Abstract

**Introduction:**

Total femur replacement is an option instead of amputation for extensive bone tumour or after revision surgery with a massive bone loss. Over a long period of time the patients may need revision surgery, and this might affect the functional outcome. We reviewed all consecutive total femur replacements done for primary and revision surgery of primary bone tumours in our centre to evaluate the long-term functional outcome and survival.

**Materials and methods:**

All patients who had total femur resection and reconstruction with modular endoprosthesis replacement in our centre from June 1997 to May 2022 were reviewed. The respondents were surveyed through WhatsApp using google form which was translated into Bahasa Malaysia based on the Musculoskeletal Tumour Society Scoring System (MSTS). The data were presented as descriptive data on the final survival of the limb and prosthesis.

**Results:**

Ten patients underwent total femur replacement. There were eight osteosarcoma, one giant cell tumour and one chondromyxoid fibroma. Three patients with osteosarcoma succumbed to pulmonary metastases; all had good early post-operative functional outcomes without local recurrence. Seven patients were available for long term evaluation of function with a mean follow-up of 17.6 years (ranged 10-25 years). Four patients with total femur replacement had good functional outcomes (60-80%) without revision with 10-25 years follow-up. Three patients experienced acetabulum erosion and chronic pain that required early hip replacements. Two of them were complicated with superior erosions and bone loss and subsequently were managed with massive reconstruction using cemented acetabulum cage reconstruction. The other has diabetes mellitus with chronic infection following revision of distal femur endoprosthesis to total femur replacement and subsequently underwent limited hemipelvectomy after 14 years.

**Conclusion:**

Total femur replacement offers a good long term functional outcome and prosthesis survival and is a favourable option for limb salvage surgery.

## Introduction

Total femur replacement is an option for extensive primary bone tumours. Stable reconstruction after resection is impossible because of the limited length of the remaining bone due to extensive bone loss to achieve a safe oncologic margin or after a revision surgery. The lower limbs’ functional outcome is positive, despite major resection and double joint reconstruction^1-3^. The newer generation of endoprostheses, with modularity, was able to maintain limb length and joint size for optimal muscle balance, patellar tracking, and adductor function for optimum limb functional outcome.

With a modest number of revision surgeries or delayed amputations, a long-term prosthetic survival rate of 80% was achieved after 10 years^2-4^.

## Materials and Methods

All patients who had total femur resection or a revision surgery and reconstruction with modular endoprosthesis replacement in our centre from June 1997 to May 2022 were reviewed. The epidemiological data, surgical indication, and follow-up in terms of surgical revision and patients’ functional outcome were evaluated. For the final functional outcome, all respondents were surveyed through WhatsApp and google form in Bahasa Malaysia using the Musculoskeletal Tumour Society Scoring system (MSTS)5. The data were presented as descriptive data on the final survival of the limb and prosthesis.

## Results

**Fig 1:**
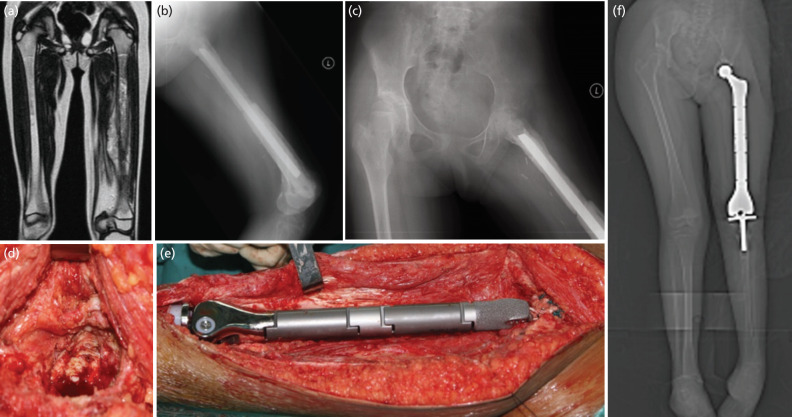
Case 6. A 8-year-old girl with diaphyseal osteosarcoma crossing the distal femur physis with bone marrow extension to the trochanter was initially managed with resection of the entire femur with allograft reconstruction. Four years later, the entire construct was converted to total femur endoprosthesis at her age of 12 due to severe acetabulum erosion and pain. The limb length discrepancy was managed by contralateral all physeal epiphysiodesis. She was ambulating pain-free without support with 3cm shortening 12 years following TFR surgery. (a) MRI showed extensive marrow involvement of the entire femur, (b) total femur allograft reconstruction, (c) acetabulum erosion on pelvic radiograph, (d) Operative photo showed acetabulum cartilage erosion, € Total femur endoprosthesis with hip replacement, (f) lower limb scanogram showed total femur replacement with limb shortening of 2cm.

Ten patients underwent total femur replacement. There were eight osteosarcoma, one giant cell tumour and one chondromyxoid fibroma. Seven of them with osteosarcoma had primary total femur replacements due to skip lesions and extensive bone marrow involvement. The other two cases with giant cell tumour and chondromyxoid fibroma of distal femur had proximal stem loosening and fractures that were revised to a total femur replacement. Another child which initially had total femur allograft reconstruction was converted to a cemented hip resurfacing total femur replacement due to hip pain and unstable knee (Fig. 1). Three patients with osteosarcoma succumbed to pulmonary metastases; all had good early post-operative functional outcomes without local recurrence.

Seven patients were available for evaluation of function with a mean follow-up of 17.6 years (ranged 10-25 years). Four had good functional outcomes (60-80%) without revision with 10-25 years follow-up. One had metachronous lesion to the contralateral distal femur and underwent resection and endoprosthesis replacement. The same patient also underwent a revision of distal femur endoprosthesis due to knee component failure, but the total femur was stable for 21 years with a MSTS score of 63%. Three patients experienced acetabulum erosion and chronic pain that required early hip replacements. Two patients were complicated with superior erosions and bone loss and subsequently were managed with massive reconstruction using cemented Gap II (Stryker Howmedica) reconstruction. Tripolar hip endoprothesis reconstruction was attempted; however, the acetabulums were reconstructed with cemented restraint cups due to intra-operative instability. The final reconstruction was stable at 5 and 10 years.

**Fig 2:**
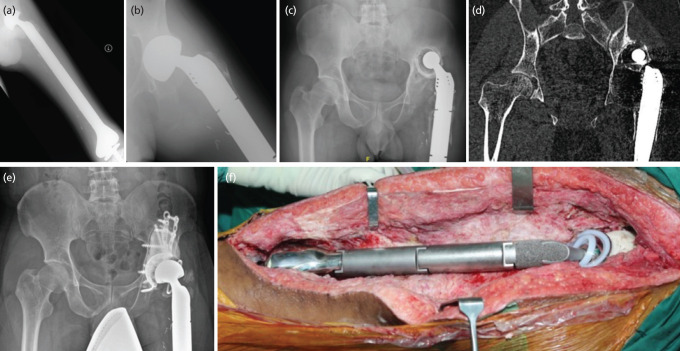
Case 3. A 16-year-old boy, diagnosed with distal femur osteosarcoma and medullary extension to the trochanteric region. Wide resection and total femur endoprosthesis were done at the age of 16-year-old. The bipolar femoral head had to be converted to cemented liner due to early cartilage erosion. He came eight years later with limb shortening and an unstable hip due to superolateral erosion of the acetabulum. The hip was reconstructed with cemented Gap II anti protrusio cage and constraint cup for the stability of the hip. After ten years of revision, he is now ambulating pain-free and has a stable hip. (a, b) Total femur endoprosthesis with bipolar head showed acetabular erosion. (c, d) Radiograph and CT scan showed polyethylene liner had migrated superiorly with significant bone loss, (e) Cemented anti protrusio cage with constraint cup. (f) Intra-operative photo showed cemented constrained cup with bipolar articulation.

One patient was satisfied with his outcome, even though he had a 2cm shortening and a limited range of the knee motion of 0-70° (Fig. 2). Another patient had a poor outcome due to reduced hip function and a stiff knee with motion limited to 0-30° and needed a single stick for walking. An obese patient with a BMI of 42 and diabetes mellitus was complicated by chronic infection following revision of distal femur endoprosthesis into a total femur replacement and subsequently underwent limited hemipelvectomy after 14 years. He was ambulatory with a single crutch with total femur replacement and used a wheelchair after amputation, with a MSTS score of 26% (Table I, II).

**Table I:** Series of 10 cases with long term outcome for total femur replacement

No	Age	Sex	Primary Disease	Indication Total Femur	Implant	Free Flap	Follow-up (years)	Oncology outcome	Long term complication	Failure Henderson	Reoperation (no of surgery after TFR)	Final Final
1	17	M	DF OS	Skip lesion neck of femur	HMRS	Lat Dorsi	25	DFS	Nil	nil	nil	Full function
2	16	M	DF OS	Marrow extension to PF	GMRS	Lat Dorsi	21	SWD Lung metastasis - lobectomy Contralateral DF - WR Endoprosthesis (6 yrs)			DF knee component break at 13 yrs - change entire prosthesis Restore to GMRS	Full function
3	16	M	DF OS	Marrow extension to PF	GMRS	-	20	DFS	Cartilage erosion at 2 yrs. Loosening acetabulum liner and infection (8 yrs)	3	Conversion bipolar - cemented acetabulum Antibiotic space - Gap II constraint hip (3)	Mobile hip Stiff knee 0-700 Shortening 2cm
4	32	F	DF GCT	Loosening stem at 2 yrs with fracture - Flenderson 3	Biomet	-	19	DFS	Painful erosion hip at 8 yrs. Loosening constraint hip at 5 yrs. Loosening with superior erosion at 4 yrs	3	Revision constrain hip Mesh wire - new constraint hip - Gap II cemented constraint hip (3)	Mobile hip stiff knee 0-300
5	31	M	DF Chondromyxoid fibroma	Infected loosening DF bone loss at 3 yrs - Henderson 3	GMRS	-	16	DFS	Erosion acetabulum medial protrusion (4 yrs). Chronic infection knee. Loosening hip implant and medial protrusion (10 yrs). Diabetes mellitus	4	Meshed wire, Gap II and constraint hip Hemipelvectomy (5)	wheelchair
6	8	F	Diaphyseal OS	Involve growth plate DF and extend to trochanter Total femur allograft Flip pain after 4 yrs	GMRS		12	DFS	nil		Contralateral epiphysiodesis	Shortening 3cm
7	22	M	DF OS	Interlocking nail for midshaft femur fracture	USTAR	Lat dorsi	10	Thoracotomy for lung metastases DFS				Adductor weakness - Trandelenberg gait
8	10	F	DF OS	Marrow extension to trochanter	GMRS	Lat Dorsi	2	DOD at 2 yrs				Peroneal nerve palsy - ambulating ankle orthosis
9	35	F	DF OS	Marrow extension to trochanter	Guardian	Lat Dorsi	2	DOD at 2 yrs				Ambulating with frame
10	17	M	DF OS	Marrow extension to trochanter	GMRS		1	DOD 18 months				Full function

Abbreviations - DF: Distal Femur, OS: Osteosarcoma, GCT: Giant cell tumour, Lat Dorsi: Latissimus dorsi flap, FIMRS: Flowmedica Modular Replacement System, GMRS: Global Modular Replacement System, Biomet Oncology Salvage System, Wright 'Guardian Limb Salvage System, USTAR Limb salvage system (United Orthopaedic Corporation), DFS: disease free survival, SWD: survive with disease, DOD: died of disease

**Table II:** Detailed functional outcome based on MSTS Score

No	Musculoskeletal Tumour Society Score						
	Pain	Function	Emotion	Support	Walking	Gait	Percentage
1	5	4	5	5	5	3	80%
2	4	2	2	5	3	3	63%
3	5	4	5	5	5	5	97%
4	3	1	2	1	1	1	33%
5	4	0	1	0	3	0	26%
6	4	4	3	5	4	3	77%
7	5	1	1	5	3	3	60%
8	5	3	4	5	4	4	78%
9	5	3	4	4	4	4	75%
10	5	4	4	5	5	4	83%

## Discussion

Revision-free long-term survival was reported between 50-70% at 10 years, with the overall survival of the total femoral replacement (TFR) implant being around 80-90%^2-4^. In our study, long-term TFR survival was four out of seven and revision was mainly secondary to acetabulum erosion. The amputation surgery had a negative impact on the patient's ability to function. Total femur replacement, on the other hand, gives good early lower limb function and so is an option in limb salvage surgery in total oncological femur resection. It is also performed in revision surgery when the residual bone is insufficient to hold the prosthetic stem in place. There are a few viable options, especially in the era of precision medicine, whereby an oncological margin can be accurately determined for bone stock preservation and better functional outcomes. Wong *et al* established the safety profile of joint preservation tumour resection using image-guided navigation and custom prosthesis, with outstanding long-term functional outcome^6^. Compression cementless stem fixation [Compress® Zimmer Biomet] allows for stable short remaining bone fixation and also promotes new bone fixation and osteointegration^7^. To reduce the use of total femur replacement with two artificial joint reconstructions, allograft and other methods of bone recycling, such as extracorporeal irradiation and cryotherapy, are options^8,9^. A combination of allograft and prosthesis composite allows functional joint reconstruction and retains bone stock for future revision in the young active patients. Total femur resection and replacement can be managed with different techniques for maintaining the patients’ hip joints in all of our cases, based on current technology^8^. Cryotherapy and delayed limited replacement may be beneficial in cases of skip metastases to the femoral neck.

**Fig 3:**
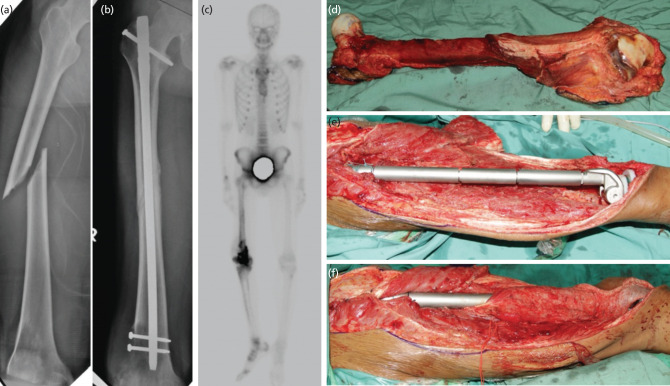
Case 7. A 23-year-old gentleman presented with a traumatic spiral fracture involving the midshaft of the right femur. Radiograph revealed the presence of an ill-defined osteolytic lesion in the distal metaphysis of the ipsilateral femur associated with cortical destruction with minimal soft tissue opacity at the posterolateral aspect of the region. He was initially treated with an interlocking nail, and the fracture united. However, there was a progression of distal femur lytic lesion, and biopsy confirmed the conventional type of osteosarcoma. He underwent four courses of chemotherapy and responded well. Total femur with distal quadriceps resection was performed to achieve a wide and adequate margin due to contamination from previous surgery. The limb was reconstructed with total femur endoprosthesis and a motorised latissimus dorsi flap. He is now 10 years post-surgery, disease-free, and ambulating without support with a 20° knee extension lag due to quadriceps weakness. (a) Traumatic oblique fracture mid-shaft femur with early lytic and rarefaction of distal femur. (b) the distal femur lytic lesion progressed but the fracture united well. (c) Bone scan showed active lesion occupied the distal femur and no evidence of distant metastases. (d, e, f) resection of the entire femur with anticipated contamination to the soft tissue and endoprosthesis replacement with latissimus dorsi flap.

Overall, medium to long-term functional outcomes ranged between fair and good, with MSTS ranging from 15-25^2-4^. Poor gait patterns secondary to lack of abductor muscle attachment to prosthesis contributed to the final functional outcome. Massive tumours with substantial intramedullary extension demand an extensive soft tissue resection to achieve oncological clearance. Reconstruction using composite tissue transfers in five patients with latissimus dorsi provides soft tissue cover around the knee joint for the stability of patella tracking and knee motion. Four out of five patients with massive tissue resection that required flaps had pulmonary metastases, and two of them had long-term survival after thoracotomy and salvage chemotherapy. One patient with a midshaft femur fracture with distal femur osteosarcoma was initially managed with an interlocking nail and responded well to chemotherapy. In wide resection of the distal femur involving the entire vastus muscles with sparing of the rectus tendon, total femur endoprosthesis together with motorised latissimus dorsi muscle enhances the lower limb function^10^. Resection of part of gluteus maximus and medius for contaminated margin over the nail entry site resulted in severe Trendelenburg gait and a fair MSTS score function of 60% (Case 7, Fig. 3)

Structural and soft tissue failure have been reported for early revision of total femur replacement. Bipolar hip endoprosthesis provides early stability with purse string capsular reinforcement, but in the long term, it redisposed to protrusio acetabuli and lateral erosion leading to chronic pain^2-4,11,12^. Three patients needed acetabulum replacement due to cartilage erosion and being symptomatic. In cases when life expectancy exceeds 10 years, resurfacing the acetabulum is advisable. Hip stability can be provided with a newer generation dual mobility component or soft tissue reconstruction with a trevira tube (polyethylene terephthalate (PET) for better abductor attachment and stability. We noticed that early revision with ordinary acetabulum linear is possible, but in cases of chronic fibrosis, the only option is a constraint cup due to intra-operative instability and poor abductor strength^2-4,11,12^. Knee component failure in total femur replacement is uncommon, rotating components place less stress on knee reconstructions, and long-term patellar erosion is not a major issue. Complete dissociation of the total femur due to poor soft tissue attachment has been reported, and reattachment of soft tissue with trevira tube attachment significantly reduces the incidence.

The main problem observed in our patient was early hip joint erosion that led to chronic pain and delayed acetabular erosion. Abductor weakness and Trendelenburg gait exert high stresses on the hip joint and hence, led to this problem. Furthermore, delayed revision surgery to the acetabulum in poorly functional abductor and extensive fibrosis significantly increases the risk of hip dislocation. Reconstruction of acetabulum defect and reinforcement with cages with constraint cup is a viable option to achieve hip stability for medium-term function.

## Conclusion

The overall survivorship in patients with extensive primary osteosarcoma had positive impact in the past few decades. However, there are still debates and uncertainties about whether or not to undergo an amputation or limb salvage surgery. The amputation surgery had negative impact on the patient's ability to function. Total femur replacement, on the other hand, offers good early lower limb function and therefore, it is a favourable option in limb salvage surgery in total oncological femur resection. Having a long-term prosthetic survival rate of 80% after 10 years with a modest revision surgery, TFR had been shown to give a good functional outcome based on MSTS scoring system and reduced the incidence of lower limbs amputation in our population.
